# Tissue and exosomal miRNA editing in Non-Small Cell Lung Cancer

**DOI:** 10.1038/s41598-018-28528-1

**Published:** 2018-07-05

**Authors:** Giovanni Nigita, Rosario Distefano, Dario Veneziano, Giulia Romano, Mohammad Rahman, Kai Wang, Harvey Pass, Carlo M. Croce, Mario Acunzo, Patrick Nana-Sinkam

**Affiliations:** 1 Department of Cancer Biology and Genetics, The Ohio State University, Columbus, OH USA; 2 Division of Pulmonary Diseases and Critical Care Medicine, Virginia Commonwealth University, Richmond, VA USA; 3 Institute for System Biology, Seattle, WA USA; 4 Department of Cardiothoracic Surgery, New York University Cancer Center, New York, NY USA

## Abstract

RNA editing in microRNAs has been recently proposed as a novel biomarker in cancer. Here, we investigated RNA editing by leveraging small-RNA sequencing data from 87 NSCLC (Non-Small Cell Lung Cancer) samples paired with normal lung tissues from The Cancer Genome Atlas (TCGA) combined with 26 plasma-derived exosome samples from an independent cohort. Using both the editing levels and microRNA editing expression, we detected deregulated microRNA editing events between NSCLC tumor and normal tissues. Interestingly, and for the first time, we also detected editing sites in the microRNA cargo of circulating exosomes, providing the potential to non-invasively discriminate between normal and tumor samples. Of note, miR-411-5p edited in position 5 was significantly dysregulated in tissues as well as in exosomes of NSCLC patients, suggesting a potential targetome shift relevant to lung cancer biology.

## Introduction

Lung cancer is the number one cause of cancer-related deaths among men and women (Cancer Fact and Figures 2017, American Cancer Society). Targeted therapy and early detection of lung cancer should be priorities and remain the most effective approaches to significantly reducing the number of deaths from the disease.

RNA editing is a widespread molecular phenomenon in metazoa^1^ that involves base substitution of nucleotides within RNA^2^. RNA editing has been observed in both coding and noncoding genes including microRNAs (miRNAs)^3,4^. The RNA editing phenomenon is further defined by nucleobase modifications, consisting of the deamination of cytidine (C) to uridine (U), and adenosine (A) to inosine (I). Inosine is, in turn, interpreted as guanosine (G) by both the splicing and the translation machineries^5^. A-to-I RNA editing events, defined as “*canonical editing sites*”, occur in coding mRNA sequences and may alter the amino acid sequence of the encoded protein, influencing both function and regulation^2,6^. This type of modification is considered to be “dynamic”, as it is not linked to DNA changes but rather to post-transcriptional events associated with a family of proteins called ADARs (Adenosine Deaminase Acting on RNA)^2,3^, which bind double-stranded RNAs (dsRNs)^7^. It has been shown that deregulation of the ADARs can contribute to the pathogenesis of human diseases^8^, including lung cancer^9^. Thus, RNA editing is emerging as a new mechanism for carcinogenesis^10,11^.

Although recent research on RNA editing has focused primarily on elucidating the functional relevance of these modifications in coding RNA, attention has also shifted towards the investigation of editing events involving non-coding RNAs (ncRNAs). In 2007, Nishikura’s group reported that RNA editing also occurs in microRNAs (miRNAs)^12^. It has been estimated that 10–20% of miRNAs undergo A-to-I editing at the pri-miRNA level^13^. While A-to-I pri- or pre-miRNA editing events may affect both the maturation and the expression of miRNAs, those occurring in the mature sequence, particularly in miRNA seed regions (MSRs), could drastically alter the spectrum of microRNA targets (“targetome”) and consequently modify their function^14^. We recently studied miRNA editing effects in a dynamic cellular context such as hypoxia and demonstrated a connection between miRNA editing and the activation of certain cellular pathways during hypoxic conditions^15^. Edited (ED) miRNA molecules may play the same role of wild-type (WT) miRNAs, with their dysregulation conferring tumor suppressor or oncogenic proprieties^16^. Indeed, it has been recently proposed that ED miRNAs could function as potential biomarkers for cancer prognosis and therapy^17–19^.

To date, the miRNA editing phenomenon has been studied by exclusively considering the editing level, which is based on the expression of the miRNA itself (Editing Level = ED miRNA/(ED miRNA + WT miRNA). Such a parameter is dependent on the expression of the WT miRNA and thus carries a bias that may impede a correct interpretation of the results. Here, we apply a new concept for miRNA editing measurement, which considers not only the editing level but also the expression of ED and WT miRNAs assessed via *reads per million reads mapped to miRNAs* (RPM). We used NSCLC as the model disease to test this approach. We examined small-RNA sequencing data from 43 Lung Adenocarcinoma (LUAD), and 44 Lung Squamous Cell Carcinoma (LUSC) samples paired with normal lung tissues provided by The Cancer Genome Atlas (TCGA) collection. Using the editing level and miRNA expression, we identified deregulation of ED miRNAs between tumor and normal samples in both LUAD and LUSC, respectively. Interestingly, this latter parameter proved to be more efficient in distinguishing normal and tumor tissues in both types of lung cancer.

Furthermore, for the very first time, we wanted to determine whether miRNA editing events occurred in circulation. To accomplish this, we analyzed small-RNA sequencing data from exosome samples from an independent cohort of NSCLC patients at different stages. We identified two ED miRNAs in circulation able to distinguish between normal and tumor sample subtypes. Interestingly, one of these circulating ED miRNAs, miR-411–5p edited in position 5, was also differentially expressed between NSCLC and normal tissue samples.

## Results

### Systematic characterization of the miRNA editing in NSCLC tissue samples

**Figure 1:**
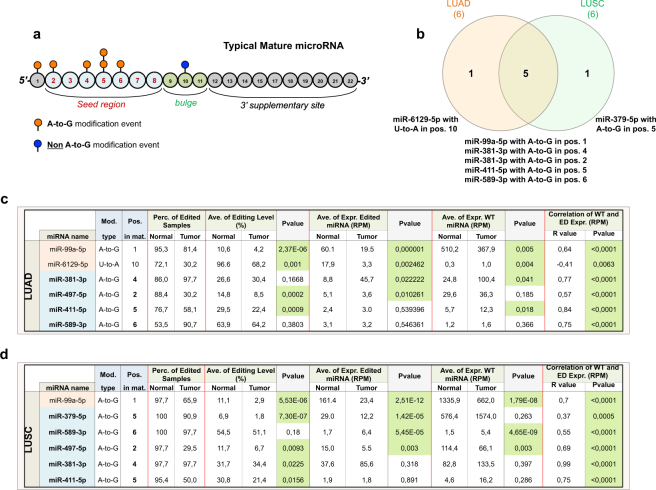
RNA editing hotspots in LUAD and LUSC tissue samples. (**a**) Diagram showing the distribution of all detected editing hotspots across miRNA nucleotide positions. (**b**) Venn diagram of RNA editing hotspots in LUAD and LUSC samples, showing that the majority of editing hotspots is shared. (**c**,**d**) Statistics for miRNA editing hotsposts and WT counterparts in normal and tumor samples for both LUAD and LUSC. Hotsposts occurring within MSRs are in *light blue*, while those outside the MSR are in *light orange*. P-values were calculated with the following methods respectively: Mann-Withney paired test for editing level; paired t-test for ED\WT miRNA expression; Pearson correlation test between WT\ED miRNA expressions. Significant P-values (P < 0.05) are in *green*.

To systematically identify such modification events (MEs) in NSCLC tissue samples, we applied the Alon-Eisenberg pipeline^20^ (see Supplementary Fig. S1 and Methods section) to TCGA-derived small RNA sequencing (sRNA-seq) data^21^, from 43 LUAD and 44 LUSC tissues paired with normal lung samples. As shown in Supplementary Data Set 1, we identied 40 and 18 high-confidence MEs in LUAD and LUSC (as defined in Methods section), respectively, which were not reported as single nucleotide polymorphisms (SNPs, considering common dbSNP build 150), nor were they labeled as somatic mutations in LUAD and LUSC cohorts. Similarly to a recent study^18^, we focused on 7 distinct high-confidence miRNA ME hotspots (as defined in Methods section), 86% of which (6 out 7) canonical A-to-G MEs. Among these miRNA ME hotspots 5 (71%) are located in miRNA seed regions (MSRs, within nucleotide positions 2–8, see Fig. 1a), 6 (86%) have been detected in previous studies (Supplementary Table S1). Noteworthy, miR-6129-5p with a U-to-A ME in position 10, and miR-379-5p with A-to-G ME in position 5 are specific for LUAD and LUSC samples, respectively, while the remaining MEs have been detected in both cancer subtypes (Fig. 1b).

Considering that the editing level depends on WT miRNA expression, as shown in Fig. 1c,d (correlation results between WT and ED), this contributes to a bias in data interpretation. To better characterize miRNA editing in NSCLC, in addition to the editing level, we elected to consider the expression of edited miRNAs measured as *RPM* (Fig. 1c,d; Supplementary Data Set 2). In light of this new parameter, unlike previous studies^18^, miR-381-3p with A-to-G ME in position 4 and miR-589-3p with A-to-G ME in position 6, were both upregulated according to their RPM values in LUAD and LUSC, respectively, while their editing levels remained unchanged (Fig. 1c,d; see also Supplementary Fig. S2a,b). There were also miRNAs with editing levels deregulated in both LUAD and LUSC, such as miR-411-5p edited with A-to-G ME in position 5. However, their expression was unaffected in both normal and tumor conditions (Fig. 1). We have verified that the editing level alone (such as in the case of miR-589-3p with ME in position 6) does distinguish between early and late stage lung cancer (Supplementary Fig. S2c).

**Figure 2:**
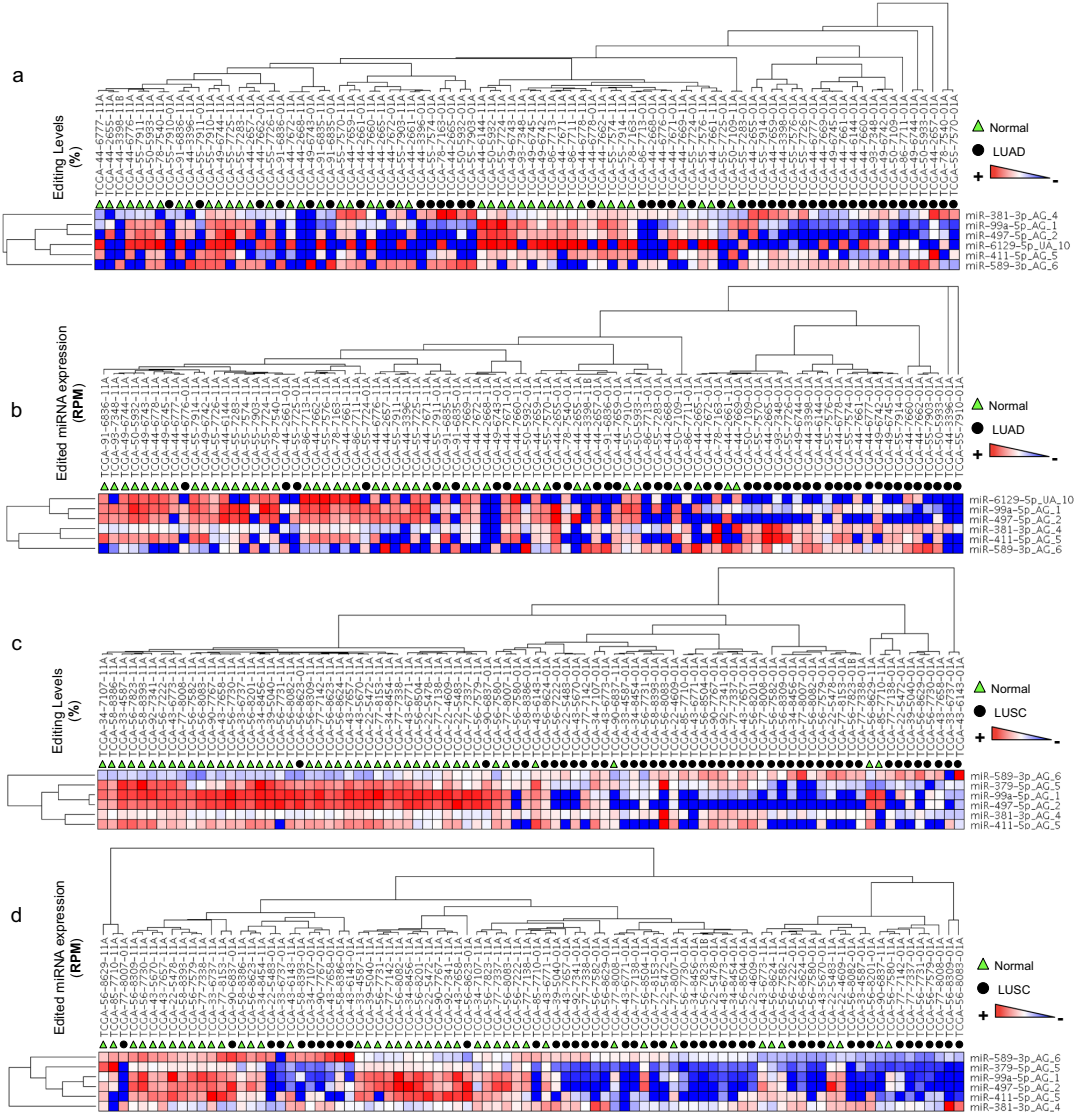
Hierarchical clustering of LUAD and LUSC tissue sample according to editing level and ED miRNA expression relative to A-to-I editing hotspots. Heat maps for miRNAs A-to-I editing hotsposts across normal and tumor samples for LUAD and LUSC, respectively. (**a**,**c**) Values considered corresponding to editing level. (**b**,**d**) Values considered corresponding to expression ED miRNAs (RPM).

By correlating the expression of the ADAR family (with ADAR1 and ADAR2 responsible for A-to-I editing events) with that of A-to-G ED miRNAs, we observed that the latter are not entirely regulated by ADARs (Supplementary Data Set 3). These findings suggest the presence of other co-factors or antagonists of the ADAR editing activity during ED miRNA biogenesis^3^. Finally, by comparing heat maps based on editing levels and RPMs respectively, we observed that *RPM* values contribute to an improved separation between normal and tumor tissues in both LUAD and LUSC histologies (Fig. 2).

### MiRNA targetome shift due to miRNA editing in NSCLC

As we have previously observed^15^, in order to assess the impact of editing on miRNA function in NSCLC, we focused on all MEs detected in LUAD and LUSC tissue samples occurring in MSRs (excluding thus miR-99a-5p with A-to-G ME in position 1 and miR-6129-5p with a U-to-A ME in position 10). By comparing the edited MSR with all WT miRNA MSRs^22^, we identified two possible scenarios (Supplementary Fig. S3): (1) the modified miRNA becomes a new miRNA (based on its MSR); (2) the edited miRNA shares the same MSR of another known miRNA.

**Figure 3:**
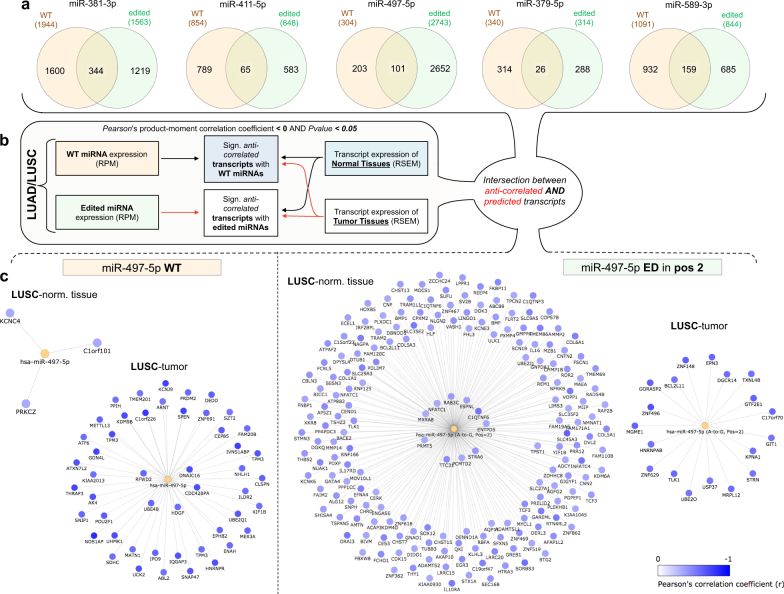
Targetome analysis of A-to-I miRNA editing in miRNA seed regions. (**a**) Predicted targetome shifting for miRNAs edited in MSRs. (**b**) Diagram of anti-correlation analysis between WT/ED miRNAs and all expressed transcripts across all samples in both LUAD and LUSC cohorts, respectively. Significant anti-correlated transcripts were considered based on a negative Pearson’s product-moment correlation coefficient and P-value < 0.05. (**c**) Diagrams of targetome shifting for WT/ED miR-497-5p in LUSC samples in both normal and tumor conditions. The miRNA targetome results as the intersection of the set of predicted targets with the set of significantly anti-correlated transcripts.

In order to elucidate the target mRNA shifting between WT and ED miRNAs, we performed a robust binding site prediction analysis on 3′-UTRs of human mRNA transcripts using a consensus of four target prediction tools (Fig. 3a and Supplementary Data Set 4): miRanda^23^, TargetScan^24^, PITA^25^ and miRiam, our in-house tool in its enhanced version^26,27^. As previously established, we employed target prediction tools when investigating the targetome of A-to-I edited miRNAs by considering inosine as guanosine^12,14^. Indeed, inosine can bind cytidine similarly to guanosine, while binding uridine in a weaker manner. Unlike guanosine, inosine can also bind to adenosine, albeit weakly^28^.

To further validate a targetome shift in the setting of miRNA editing, we performed an anti-correlation analysis (considering negative Pearson’s product-moment correlation coefficients with P-value < 0.05) between the expression of ED/WT miRNAs, respectively, and of all genes in LUAD/LUSC tumor tissues and normal adjacent tissues from the TCGA (Fig. 3b and Supplementary Data Set 4). We analyzed 18 and 37 total RNA-seq paired tumor and normal samples associated with the cohort of sRNA-seq data in both LUAD and LUSC, respectively.

Finally, for each ED/WT miRNA, significantly anti-correlated genes were intersected with a set of predicted targets (Fig. 3a,b and Supplementary Data Set 4), as specified above. In the case of miR-497-5p with ME in position 2, there were specific targetome expression shifts for both WT and ED miRNAs, respectively, in normal as well as tumor tissues in LUSC samples (Fig. 3c and Supplementary Data Set 4). A substantial cardinality difference was observed in the sets of expressed targets for WT/ED miR-497-5p, with the WT target set increasing in tumor compared with normal, while the opposite trend was observed for the ED counterpart (Fig. 3c). This phenomenon was similarly noted for the other WT/ED miRNA pairs (Supplementary Data Set 4).

### miRNA-sequence modifications in plasma-derived exosome NSCLC samples

**Figure 4:**
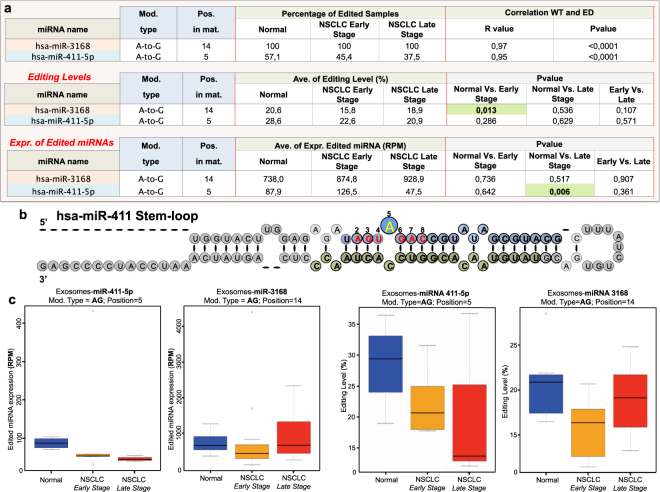
A-to-I miRNA editing hotspots in plasma-derived exosome NSCLC samples. (**a**) Statistics for miRNA editing hotsposts and WT counterparts in normal and tumor (early/late stage) plasma-derived NSCLC samples. Hotsposts occurring within MSRs are in *light blue*, while those outside the MSR are in *light orange*. Pvalues were calculated with the following methods respectively: Mann-Withney paired test for editing level; paired t-test for ED\WT miRNA expression; Pearson correlation test between WT\ED miRNA expressions. In significant pvalues (P < 0.05) are in *green*. (**b**) Diagram of stem-loop nucleotide sequence for miR-411. The -5p mature sequence is highlighted in *blue*, while in *green* is the -3p. Seed sequence nucleotides are indicated in red, with the editing site highlighted in *yellow*. (**c**) Box-plot diagrams of expression values (RPM) and editing levels for both edited miRNAs detected across normal, early and late stage NSCLC samples.

In the current study, for the very first time, we elected to investigate miRNA editing events in circulation, specifically within plasma-derived exosomes from 26 human samples (7 normal, 11 NSCLC early stage and 8 NSCLC late stage - see Supplementary Table S2). As shown in Supplementary Data Set 5, we detected 29 high confidence MEs (as defined in Methods section) in plasma-derived exosome samples, which were not reported as SNP (dbSNP build 150). For the downstram analysis, we focused on two high-confidence miRNA ME hotsposts (as defined in Methods section), both A-to-G MEs, present in exosomes from normal, as well as early and late stage NSCLC patients (Fig. 4a and Supplementary Data Set 5), and verified that both are not reported as common SNPs (dbSNP build 150). Most importantly, it is noteworthy that miR-411-5p with ME in position 5 (Fig. 4b) was also present in tissue (Fig. 1c,d) in both its WT and ED forms. Specifically, as opposed to what was observed in tissue samples, there was a significant downregulation of ED miR-411-5p expression between normal and late stage NSCLC (Fig. 4c). miR-3168 with ME in position 14 was present only in exosomes from all samples (Fig. 4a,c). There was a significant decrease in the editing level for ED miR-3168 between normal and early stage lung cancer. Finally, despite there being a strong positive correlation between the WT/ED forms of both miRNAs, as shown in Fig. 4a (correlation results; see also Supplementary Fig. S4), there was no significant difference in expression for the WT forms between normal and tumor patient samples (see Supplementary Data Set 6). This indicates that the machinery that governs the export of miRNAs to extracellular space in tumor conditions may discriminate ED miRNAs differently. Further studies are warranted to validate this hypothesis.

## Discussion

With the advent of high throughput platforms to better characterize the human genome, we have observed progress in the development of biomarkers and targeted therapies. In fact, technologies such as NGS have become essential in the genome-wide identification and investigation of the sequence integrity of ncRNAs^29^. These relatively new molecules have the potential to alter molecular functions in many human diseases, including cancer^16^. Recently, the focus has shifted to post-transcriptional modifications, such as RNA editing. The molecular phenomenon of RNA editing is a dynamic mechanism involving sequence alteration of primary RNA transcripts, both coding, and non-coding^3^, such as microRNAs (miRNAs). As recently described by Paul *et al*.^30^, during miRNA biogenesis, a suggestive crucial role for ADAR2, along with specific sequence and structural requeriments of the pre-miRNAs, is necessary for the editing phenomenon to take place. Moreover, miRNA editing provides molecular stability to the miRNA hairpin, across species in a tissue specific manner, as reported by Gallego *et al*.^31^. miRNAs are also increasingly being recognized as potential noninvasive biomarkers of disease. The fundamental role of the miRNA seed sequence in the miRNA–mRNA interaction is well recognized^32^. As previously observed^14,15,33^, a single modification event in the miRNA seed region can change the targetome of a miRNA and thus its function. Recently, Velazquez-Torres *et al*. how single miRNA editing event in seed region can prevent melanoma progression^34^.

For this reason, miRNA MEs have emerged as potential biomarkers for cancer prognosis and therapy^17–19^.

Here, we sought to investigate miRNA editing in NSCLC, exploring for the very first time the occurrence of this process also in circulation. So far, miRNA editing has been determined based on the editing level as the standard for measurement^18^. The editing level is dependent on the expression of the WT miRNA, providing a bias that may affect the interpretation of the results. In some cases, the miRNA editing level is high (e.g. more than 20%) while the absolute expression of the ED miRNA is very low (e.g. less than 1 RPM). This would suggest that the impact of the ED miRNA on the targetome is minimal, when considering the editing site within the MSR. In light of all this, here we elected to apply the expression of ED and WT miRNAs using *RPM* as an additional unit of measurement to assess miRNA editing activity. Integration of RPM in our measurement resulted in some interesting findings. For example, despite the significant correlations (mostly positive) between WT and ED miRNAs, there were cases in which the ED miRNA was deregulated as opposed to its WT counterpart and vice versa (Fig. 1c,d). These findings were not previously observed using only the editing level. This indicates that ED and WT miRNAs, despite originating from the same pre-miRNA, may be regulated distinctly^3^. Generally, our results in NSCLC tissues indicate that miRNA editing events are deregulated between normal and tumor tissues when applying both editing levels and RPM expression values. In addition, we observed an important difference between the two parameters when evaluating differential miRNA editing activity. For example, our analysis revealed that ED miR-381-3p (with A-to-G ME in position 4) and ED miR-589-3p (with A-to-G ME in position 6) were both upregulated according to their RPM values in LUAD and LUSC samples, respectively, as opposed to their editing levels which remained unaffected. Heat maps revealed that normal and tumor samples hierarchically clustered when using editing levels or RPM values. RPM values allowed for a more efficient separation of the two NSCLC types. On the other hand, we detected deregulated editing levels of a set of miRNAs in both LUAD and LUSC samples while showing unaffected expression levels. One such example is miR-411-5p edited with A-to-G ME in position 5. In particular, we detected a significant downregulation of ED miR-411-5p by RPM between exosomes from patients with late stage NSCLC and controls. However, the overall miR-411-5p editing levels remained unchanged. We also observed a significant downregulation of ED miR-411-5p in tissues but by editing levels alone. Our findings suggest the importance of taking into consideration both parameters when analyzing RNA editing.

Over the last several years, EVs have received a great deal of attention as harbingers of genetic material that may be freely exchanged between cells and their environment. miRNAs are present in several EV forms including exosomes^35,36^. Here, we have demonstrated that miRNAs undergo post-transcriptional modifications such as editing in lung cancer, and that modifications are evident in both primary tissues and circulating exosomes. Such modifications have the potential to induce shifts in miRNA targets thus altering downstream signaling. Furthermore, our study highlights the complexities of quantification and the importance of taking into consideration both absolute editing levels and RPM. While still early, we would propose that post-transcriptional modifications in miRNAs within both tissues and circulation could both serve as potential novel biomarkers and provide additional insights into the pathogenesis of cancers.

## Methods

### Data sets

We downloaded the miRNA sequencing BAM files of 43 LUAD and 44 LUSC paired with normal lung tissue samples via GDC portal (https://gdc-portal.nci.nih.gov), after obtaining authorization from the data access committee (DBGap Project ID: 11332). Before the detection analysis, we extracted raw, unaligned read sequences and quality scores (FASTQ format) from the BAM files by using the *bamtofastq* function from *bedtools* package (http://bedtools.readthedocs.io/en/latest/index.html).

### Human plasma samples

26 frozen plasma samples were received as a generous gift from the NYU plasma bank (courtesy of H.I. Pass MD, IRB approved protocol) and grouped into the following three categories: 7 control smokers, 11 early stage NSCLC, and 8 late stage NSCLC. A total of 190 μl of plasma was taken from each sample for exosome isolation.

### Exosomes isolation from human plasma

EVs were isolated using the Izon qEV size exclusion column (Izon qEV, iZON science, Cambridge MA, see Supplementary Fig. S5). 190 uL of plasma (centrifuged for 10 minutes at 10,000 × G) was added to the column membrane, followed by 310 uL of PBS. PBS was then added in 500 uL increments for a total of 32 fractions. The fractions were run on a NuPAGE Bis-Tris gel to evaluate protein contamination. Fractions 7–9 were combined as the EV fraction and fractions 12–32 were combined as the depleted fraction. Both the EV and depleted fractions were concentrated to ~150 uL using an Amicon Ultra-4 10 K centrifugal filter (Millipore cat#UFC801024). Total RNA from all 3 fractions (whole plasma, EVs and EV-depleted) was isolated using the Qiagen miRNeasy kit (Qiagen) and eluted twice with 30 uL H2O.

### Small RNA sequencing

RNA was isolated from 100 uL of plasma using the Qiagen miRNeasy Mini prep kit and eluted from the binding column in 15 uL H2O. The quality and quantity of RNA was checked with Bioanalyzer. The small RNA sequencing library was constructed with NEBNext library construction kit following manufacture’s instruction. The library with proper insert size was selected by using PippinHT from Sage Scientific. For pooling samples, NEBNext Library Quant Kit was used to quantify the library and equimolar amount of libraries from different samples was pooled for sequencing using Illumina® NextSeq 500 (50 bp single-end reads were generated).

### Detection and analysis of miRNA modification events

The detection and quantification analysis of modification events from sRNAseq data were based on two principal phases: (1) detection of miRNA editing sites by applying the Alon-Eisenberg pipeline and (2) differential expression analysis of miRNA editing hotspots (see Results section).

Briefly, we filtered all reads according to a Phred quality ≥20, with at most three positions with a lower quality. In addition, we removed sequences identified as 5′ or 3′ adaptors. Subsequently, we also removed reads whose length did not fall within the typical length range for a mature miRNA (16–27 bases).

Subsequently, all filtered reads were aligned to the human genome (hg19) employing the Bowtie software^37^, allowing for one mismatch at most and trimming the last two bases of the read^38^ (Bowtie parameters: -e 50 -a -m 1 -trim3 2 -best -strata). All the reads mapped to the known pre-miRNA sequences were considered for downstream analysis. Finally, all miRNA MEs with the mismatch base quality score ≥30 and a Bonferroni-adjusted P-value < 0.05, were taken into account for downstream analysis. The expression of each filtered miRNA MEs was calculated in RPMs. Among miRNA MEs, we have selected those which we consider to be *high confidence miRNA editing sites*, namely, having geometric mean of ED miRNA expression >1 RPM and geometric mean of editing level ≥5, in at least one condition (normal or tumor). Finally, we identified as *miRNA editing hotspots* all high confidence miRNA editing sites present in ≥50% of samples, in at least one condition (tumor or normal).

### Targetome shifting analysis

We predicted binding sites for A-to-I ED miRNA in MSR and its WT counterpart on the whole 3′UTR-ome (UCSC.hg19) through a consensus of four miRNA target predictors: PITA (v6.0)^25^, TargetScan (v7.1)^24^, miRanda (v3.3a)^23^, and the enhanced version of miRiam^26,27^. In particular, we used the standard parameters for PITA, TargetScan and miRanda, while we configured miRiam to detect canonical binding sites only, allowing no mismatches in the seed (e.g. wobble pairs).

### Statistics

Statistical significances were calculated by using *stats* R package. miRNA editing hotspot (see Results section), P-values were calculated with: Mann-Withney paired test to determine the significance in the differential editing level; paired t-test for ED\WT miRNA differential expression analysis. Pearson’s correlation for the expression of ED/WT miRNAs, respectively, and of all transcripts in LUAD/LUSC normal and tumor samples, was calculated by using the function *cor*.*test* in the *stats* R package. Venn diagrams were created by using InteractiVenn^39^. Network diagrams showing the predicted and anti-correlated genes for ED/WT miR-497-5p in LUSC samples were created by combining *JQuery* and *Vis*.*js*.

### Data availability

All rawdata that support the findings of this study have been submitted to the NCBI Gene Expression Omnibus (GEO; https://www.ncbi.nlm.nih.gov/geo/) under accession number GSE114711.

## Electronic supplementary material

Supplementary Information

Supplementary Data Set 1

Supplementary Data Set 2

Supplementary Data Set 3

Supplementary Data Set 4

Supplementary Data Set 5

Supplementary Data Set 6
