# Supplementary material for: Tissue and exosomal miRNA editing in Non-Small Cell Lung Cancer
Source: Sci Rep. 2018 Jul 5;8:10222. doi: 10.1038/s41598-018-28528-1 (PMC6033928; doi:10.1038/s41598-018-28528-1)
Supplement: Supplementary file 1 — Supplementary Information [file 41598_2018_28528_MOESM1_ESM.docx]

**Tissue and exosomal miRNA editing**

**in Non-Small Cell Lung Cancer**

**Giovanni Nigita**,^1^ **Rosario Distefano**,^1^ **Dario Veneziano**,^1^ **Giulia Romano**,^2^ **Mohammad Rahman**,^2^ **Kai Wang,**^3^ **Harvey Pass,**^4^ **Carlo M. Croce**,^1^ **Mario Acunzo,**^2, *^ and **Patrick Nana-Sinkam**^2, *^

^1^Department of Cancer Biology and Genetics, The Ohio State University, Columbus, OH, USA

^2^Division of Pulmonary Diseases and Critical Care Medicine, Virginia Commonwealth University, Richmond, VA, USA

^3^Institute for System Biology, Seattle, WA, USA

^4^Department of Cardiothoracic Surgery, New York University Cancer Center, New York, NY, USA

* Corresponding authors

Mario Acunzo and Patrick Nana-Sinkam contributed equally to this work.

Correspondence and requests for materials should be addressed to M.A. (email: *Mario.Acunzo@vcuhealth.org*) or to SPN (email: *Patrick.Nana-Sinkam@vcuhealth.org*)

**
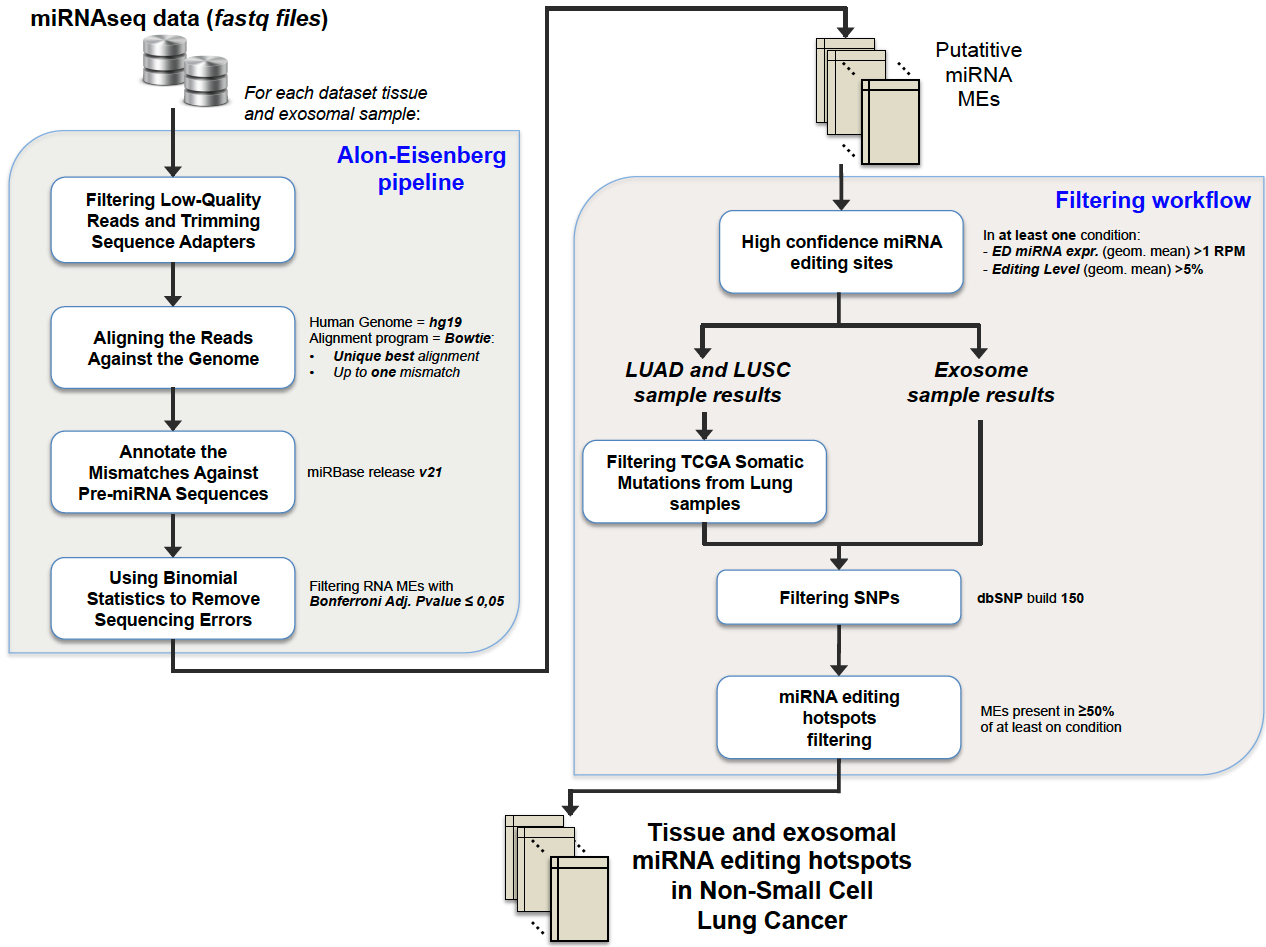
**

**Supplementary Figure S1. miRNA editing hotspots detection workflow.** Pipeline to detect miRNA editing hotspots, with a specific reference to NSCLC tissue and exosomal samples.**
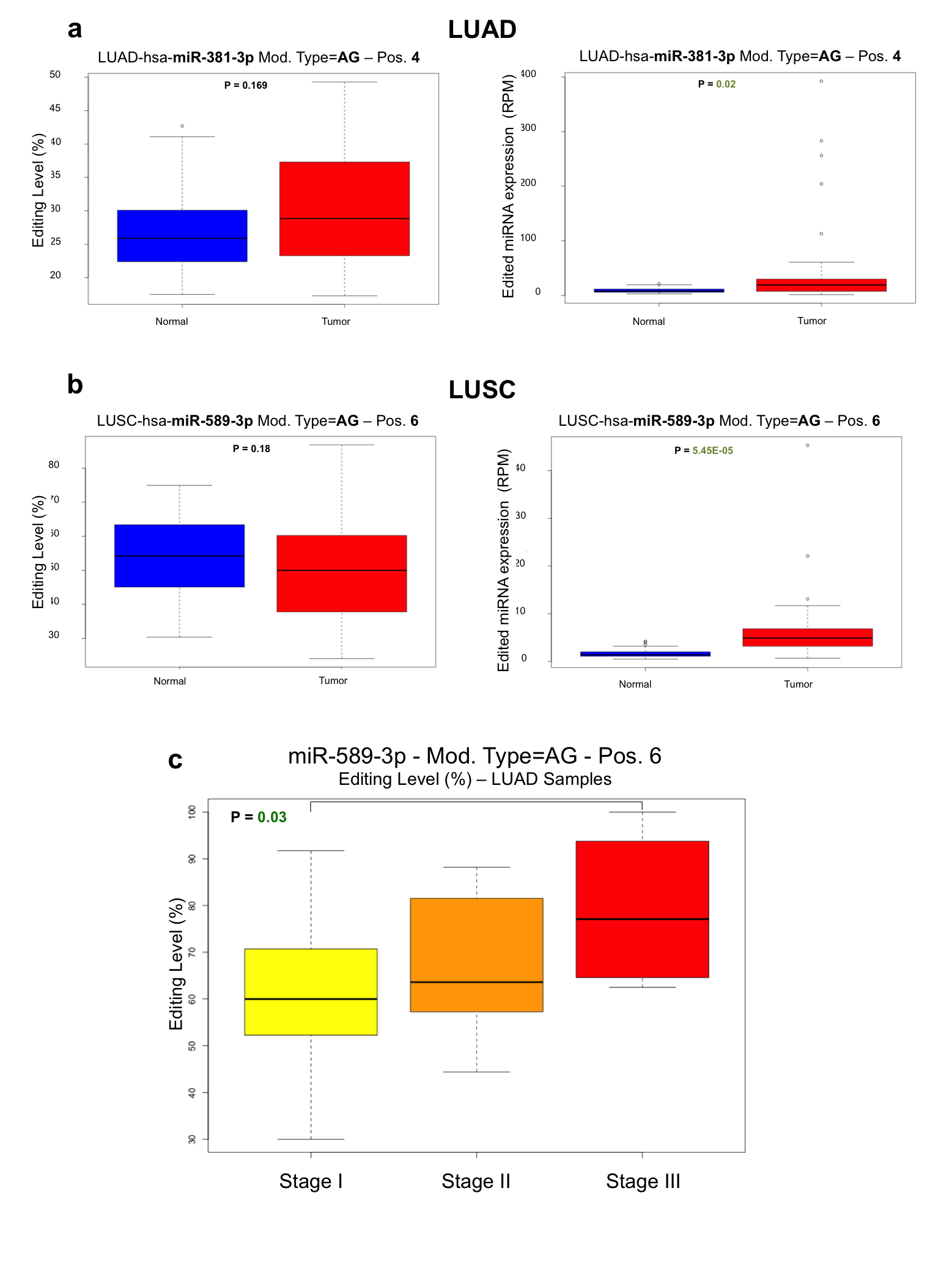
**

**Supplementary Figure S2. Boxplots for the comparison of editing level and RPM values. (**a) and (b) Comparison of editing level and ED miRNA expression (RPM) for miR-381-3p with A-to-G ME in position 4 in LUAD samples, and miR-589-3p with A-to-G ME in position 6 in LUSC samples. Significant difference (Pvalue <0.05) was recorded for ED miRNA expression, while there is no significance in differential editing level. (c) Editing levels for miR-589-3p with A-to-G ME in position 6 in LUAD samples in stages I-III. Significant difference was recorded for editing levels between stages I and III. Pvalues were calculated with the following methods respectively: Mann-Withney paired test for editing level; paired t-test for ED\WT miRNA expression.

**
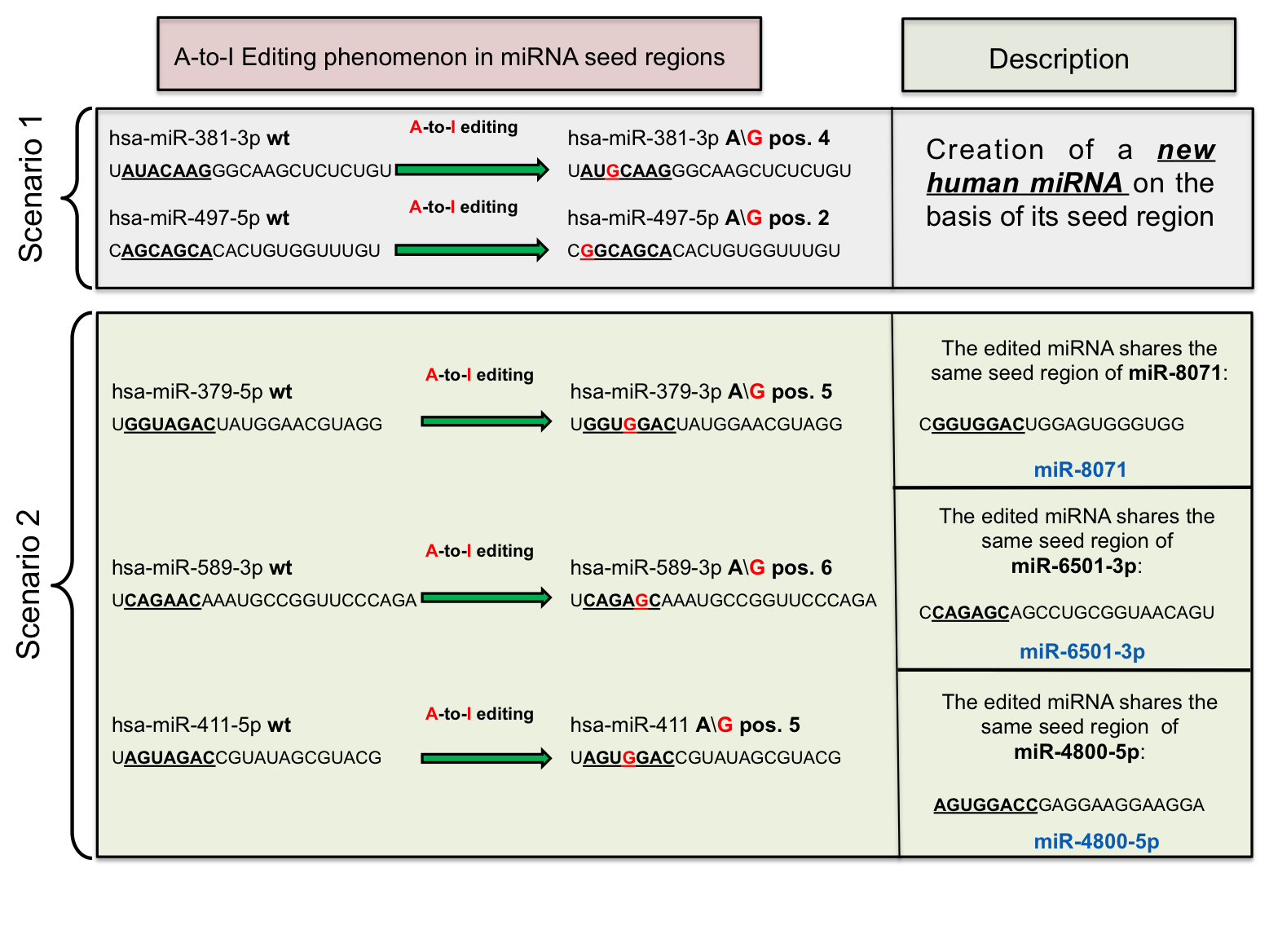
**

**Supplementary Figure S3. Diagrams of different MSR editing scenarios for five miRNA editing hotspots in NSCLC samples.** Scenario 1 depicts the case in which miRNA editing in MSR provides a novel human miRNA sequence; Scenario 2 depicts the case in which miRNA editing in MSR confers a seed sequence shared with a known human miRNA.

**
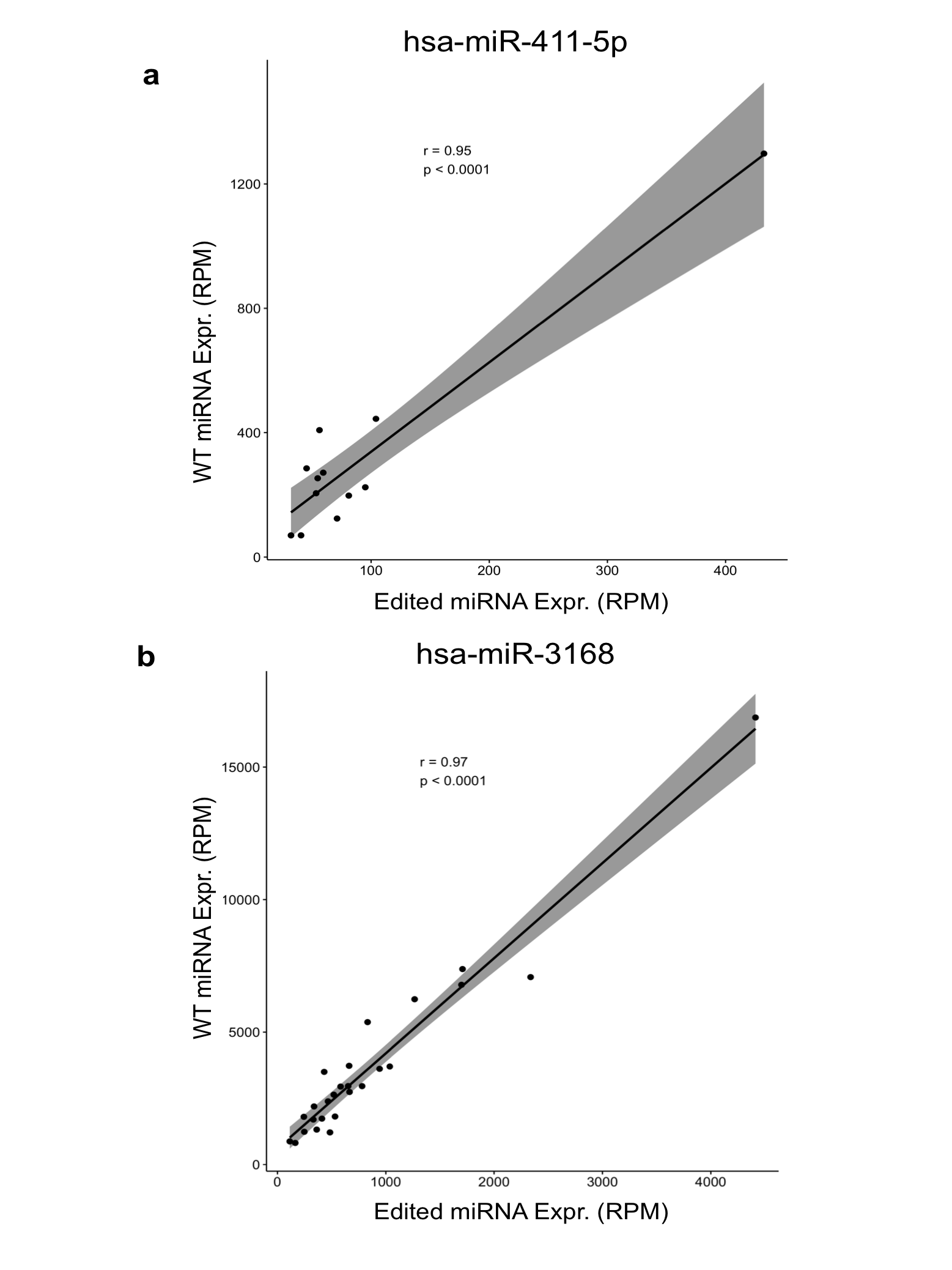
**

**Supplementary Figure S4. Expression correlation analysis between WT and ED forms for miR-411-5p and miR-3168 in plasma-derived exosome samples.** Scatter plots showing the significant (P<0.05) correlation between the expressions of WT and ED forms for miR-411-5p (a) and miR-3168 (b) in plasma-derived exosome samples. Pearson correlation test between WT\ED miRNA expressions.


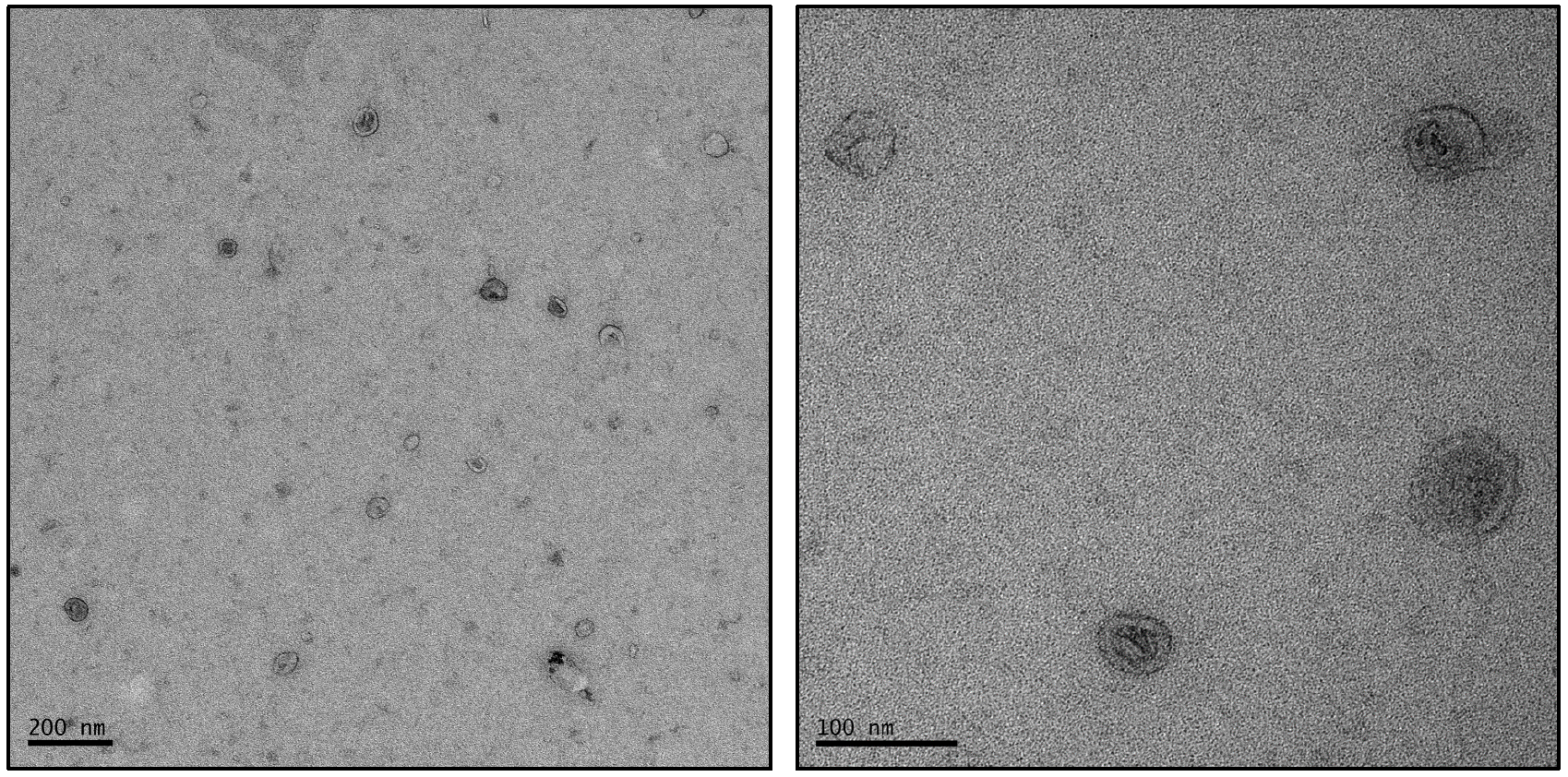


**Supplementary Figure S5. TEM images for exosomes.** TEM images for exosomes after isolation with qNano (iZono, Cambridge, MA).

| **Pre-miRNA** | hsa-mir-379 | hsa-mir-381 | hsa-mir-411 | hsa-mir-497 | hsa-mir-589 | hsa-mir-99a |
| --- | --- | --- | --- | --- | --- | --- |
| **Mature miRNA** | hsa-miR-379-5p | hsa-miR-381-3p | hsa-miR-411-5p | hsa-miR-497-5p | hsa-miR-589-3p | hsa-miR-99a-5p |
| **Pre-miRNA Pos.** | 10 | 52 | 20 | 25 | 66 | 13 |
| **Mature miRNA Pos.** | 5 | 4 | 5 | 2 | 6 | 1 |
| **Modification Type** | A-to-G | A-to-G | A-to-G | A-to-G | A-to-G | A-to-G |
| **RNA sequence** | **UGGUAGACUAUGGAACGUAGG** | **UAUACAAGGGCAAGCUCUCUGU** | **AUCAACAGACAUUAAUUGGGCGC** | **CAGCAGCACACUGUGGUUUGU** | **UCAGAACAAAUGCCGGUUCCCAGA** | **AACCCGUAGAUCCGAUCUUGUG** |
| **Sequence Length** | 21 | 22 | 23 | 21 | 24 | 22 |
| **PMID Source** | 16594986, 18684997, 22499667, 26449202, 27229138, 28550310, 29165639, 29233923 | 20413612, 22499667, 26449202, 27229138, 28550310, 29165639, 29233923 | 18684997, 22499667, 25582055, 26449202, 27229138, 28550310, 29165639, 29233923, 29445025 | 20413612, 22499667, 25582055, 27229138, 28550310, 29165639, 29233923 | 22499667, 25582055, 26449202, 27229138, 27298257, 28550310, 29165639,­ 29233923, 29267965 | 16594986, 18684997, 22499667, 25582055, 26449202, 28550310, 29165639, 29233923 |

**Supplementary Table S1. Known modification events hotsposts.** miRNA editing hotspots are in highlighted in red in the RNA sequence. For each miRNA editing hotspot the Pubmed ID source is reported.

|  | **Patient ID** | **SEX** | **Age** | **Nodule Type** |
| --- | --- | --- | --- | --- |
| **CTRL Patients** | C1 | F | 55 | No NODULES |
|  | C2 | F | 52 | No NODULES |
|  | C3 | M | 76 | No NODULES |
|  | C4 | M | 54 | No NODULES |
|  | C5 | M | 72 | No NODULES |
|  | C6 | F | 56 | No NODULES |
|  | C7 | M | 78 | No NODULES |
|  | **Patient ID** | **SEX** | **Age** | **Pathological stage** |
| **NSCLC Early Stage** | E1 | F | 67 | IA |
|  | E2 | F | 67 | IA |
|  | E3 | M | 60 | IB |
|  | E4 | F | 64 | IA |
|  | E5 | F | 65 | IA |
|  | E6 | F | 71 | IB |
|  | E7 | M | 64 | IA |
|  | E8 | F | 69 | IA |
|  | E9 | M | 60 | IA |
|  | E10 | F | 70 | IA |
|  | E11 | M | 51 | IB |
| **NSCLC Late Stage** | L1 | F | 60 | IV |
|  | L2 | F | 48 | IV |
|  | L3 | F | 82 | IV |
|  | L4 | F | 64 | IV |
|  | L5 | F | 66 | III |
|  | L6 | M | 67 | III |
|  | L7 | M | 62 | II |
|  | L8 | F | 60 | IV |

**Supplementary Table S2. Patient characteristics.** Description of NSCLC early and late stages as well as control patients.
